# Selenium-binding protein 1 (SELENBP1) is a copper-dependent thiol oxidase

**DOI:** 10.1016/j.redox.2023.102807

**Published:** 2023-07-04

**Authors:** Thilo Magnus Philipp, Leon Gernoth, Andreas Will, Maria Schwarz, Verena Alexia Ohse, Anna Patricia Kipp, Holger Steinbrenner, Lars-Oliver Klotz

**Affiliations:** a Institute of Nutritional Sciences, Nutrigenomics Section, Friedrich Schiller University Jena, Jena, Germany; b Institute of Nutritional Sciences, Department of Nutritional Physiology, Friedrich Schiller University Jena, Jena, Germany

**Keywords:** MTO, Methanethiol, Volatile sulfur compounds, VSC, H_2_S, H_2_O_2_

## Abstract

Selenium-binding protein 1 (SELENBP1) was reported to act as a methanethiol oxidase (MTO) in humans, catalyzing the conversion of methanethiol to hydrogen peroxide, hydrogen sulfide and formaldehyde. Here, we identify copper ions as essential to this novel MTO activity. Site-directed mutagenesis of putative copper-binding sites in human SELENBP1 produced as recombinant protein in *E. coli* resulted in loss of its enzymatic function. On the other hand, the eponymous binding of selenium (as selenite) was no requirement for MTO activity and only moderately increased SELENBP1-catalyzed oxidation of methanethiol. Furthermore, SEMO-1, the SELENBP1 ortholog recently identified in the nematode *C. elegans*, also requires copper ions, and MTO activity was enhanced or abrogated, respectively, if worms were grown in the presence of cupric chloride or of a Cu chelator. In addition to methanethiol, we identified novel substrates of SELENBP1 from the group of volatile sulfur compounds, ranging from ethanethiol to 1-pentanethiol as well as 2-propene-1-thiol. Gut microbiome-derived methanethiol as well as food-derived volatile sulfur compounds (VSCs) account for malodors that may contribute to extraoral halitosis in humans, if not metabolized properly. As SELENBP1 is particularly abundant in tissues exposed to VSCs, such as colon, liver, and lung, it appears to contribute to copper-dependent VSC degradation.

## Graphical abstract

**Figure:**
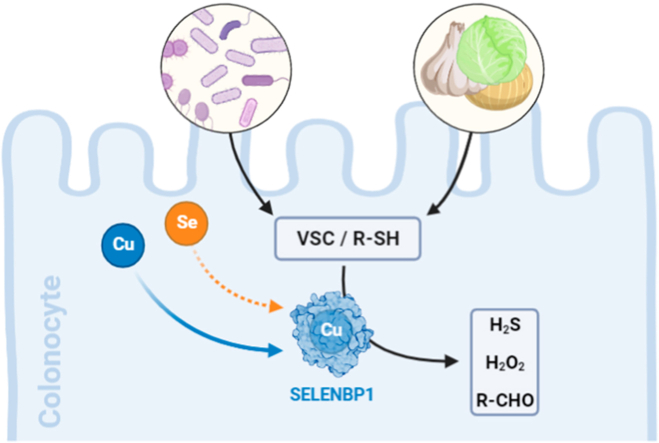

## Abbreviations

METTL7B: methyltransferase-like protein 7B
MGL: methionine gamma-lyase
MT: methanethiol
MTO: methanethiol oxidase
SELENBP1: selenium-binding protein 1
SEMO-1: SELENBP1 ortholog with MTO activity
TXRF: total reflection X-ray fluorescence

## 1 Introduction

Selenium (Se) is an essential micronutrient that is mainly active in the form of selenocysteine incorporated into 25 human selenoproteins [1]. In addition, selenium-binding protein 1 (SELENBP1) was discovered in 1989 as another Se-containing protein, after injecting mice with high doses of ^75^Se-labeled selenite [2]. In humans, SELENBP1 is ubiquitously expressed, with highest levels in colon, lung and liver [3]. Numerous observations of its suppression in various tumors and its induction during terminal differentiation gave reason to the assumption that SELENBP1 may interfere with cellular proliferation, thus acting as a tumor suppressor [[3], [4], [5], [6]]. In 2018, SELENBP1 was discovered to catalyze a novel enzymatic reaction in humans, acting as a methanethiol oxidase (MTO) that converts methanethiol to hydrogen sulfide (H_2_S), hydrogen peroxide (H_2_O_2_) and formaldehyde [7]. In this regard, we recently presented a coupled *in vitro*-assay for the straightforward assessment of MTO activity: A recombinant bacterial methionine gamma-lyase (MGL) converts methionine to the volatile MTO substrate methanethiol, and subsequently, two products of the SELENBP1-catalyzed MTO reaction, H_2_S and H_2_O_2_, are detected by trapping on lead acetate paper and fluorometric measurement, respectively [8,9].

Methanethiol, a foul-smelling gas with a low odor threshold for humans, represents an important intermediate in the global biogeochemical sulfur cycle. Microorganisms, living in soil and near-surface water as well as in the human intestine, may produce methanethiol by methylation of sulfide or degradation of sulfur-containing amino acids derived from the digestion of proteins [10]. Even though gut microbiota are the major source of exposure to methanethiol in humans, there is evidence for some endogenous methanethiol production by methylation of H_2_S mediated by methyltransferase-like protein 7B (METTL7B) [11]. Furthermore, glucose and methionine, metabolites that accumulate in cancer cells, may undergo a Maillard reaction, resulting in generation of methanethiol [12]. Inability to efficiently metabolize methanethiol due to bi-allelic mutations in the human *SELENBP1* gene manifests in extraoral halitosis, bad breath [7]. In addition to the two previously described SELENBP1 mutations that result in complete loss of its MTO activity [7,9], several other single nucleotide polymorphisms (SNPs) are found in databases for the human *SELENBP1* gene (Table 1); however, their effect on the MTO function of the protein has not been explored yet.

**Table 1:** SNPs identified for *SELENBP1* and mutants generated in this work.^a^

Position	Amino acid change	SNP ID (if available)	Se binding^b^	MTO activity^b^	Cu binding^b^	Reference	Other missense changes/SNP ID (if different)
**5**^d^	Cys→Ser	─	↓	(↓)	↑	This work	
**8**^d^	Cys→Ser	─	↓	(↓)	↑	This work	–
**57**	Cys→Ser	─	=	(↓)	(↑)	This work	–
**73**	His→Phe	─	n. d. c	↓↓	=	This work	–
**74**	His→Phe	─	n. d. c	↓↓	=	This work	–
**80**	Cys→Ser	rs1043849	↓	(↑)	(↑)	This work	Cys→Arg rs762533745
**83**^e^	Cys→Ser	rs750650780	=	(↑)	=	This work	Cys→Phe rs1180081309
**137**	His→Phe	─	n. d. c	↓↓	↓	This work	His→Gln rs752623236
**140**	His→Phe	─	n. d. c	↓↓	↓	This work	His→Arg
							His→Leu rs201591413
**141**	Cys→Ser	rs371854240	=	(↑)	=	This work	Cys→Tyr
**189**	Asp→Asn	–	n. d. c	↓↓	↓	This work	–
**225**	Gly→Trp	rs758495626	n. d.	↓↓	n. d.	[7,9]	Gly→Arg
**252**	Glu→Gln	─	n. d. c	↓↓	↓	This work	Glu→Asp rs762922279
**329**	His→Tyr	─	n. d.	↓↓	n. d.	[7,9]	His→Arg
							His→Leu rs765409789

a Source: Genome Aggregation Database (gnomAD) browser, v. 2.1.1 (https://gnomad.broadinstitute.org/).

b The symbols are: ↑ (increase by approx. 50% or more); (↑) (weak increase, less than 50%); = (no effect, less than 20% change); (↓) (weak decrease, less than 50%); ↓ (decrease by approx. 50% or more); ↓↓ (decrease by approx. 90% or more); n.d., not done.

c TXRF measurements determine both Cu and Se content in parallel. Whereas Cu is present in freshly isolated SELENBP1 (see Fig. 2), Se is not (see Fig. 1). As no selenite loading of samples analyzed for changes in Cu content was performed, there was no Se detected in these samples – hence, the analysis of Se binding was “not done”.

d As Cys^5,8^ double mutant.

e As Cys^80,83^ double mutant.

Proteins with homology to SELENBP1 occur in all domains of life [13], even though MTO activity has been substantiated to date only for human SELENBP1 and its orthologs in the bacterium *Hyphomicrobium* sp. and in the model organism *Caenorhabditis elegans* (*C. elegans*) [7,8,13]. Recently, we identified and characterized the *C. elegans* ortholog of SELENBP1, SEMO-1 (SELENBP1 ortholog with MTO activity). SEMO-1-deficient nematodes not only had a decreased H_2_S production from methanethiol catabolism, but they also showed an elevated life-span and selective alterations in stress resistance, being more resistant to oxidative stress while more sensitive to high selenite concentrations [8,14].

Structural modelling of SELENBP1 based on an ortholog from the bacterium *Sulfolobus tokodaii* suggests a highly symmetrical, seven-bladed β-propeller structure (see also Fig. 1). In SELENBP1 orthologs, short motifs containing cysteine and histidine residues are highly conserved, suggesting the presence of binding sites for “soft” metal ions such as Cu or Zn ions [8,15,16]. In fact, a bacterial ortholog (from *Hyphomicrobium* sp.) was reported to require Cu to act as an MTO [11]. SELENBP1 was discovered due to its Se-binding properties [2]; nevertheless, the mode and role of the bound Se moiety is still not well characterized; covalent binding to SELENBP1 through a seleno-sulfide bond was hypothesized [3,17].

**Fig. 1:**
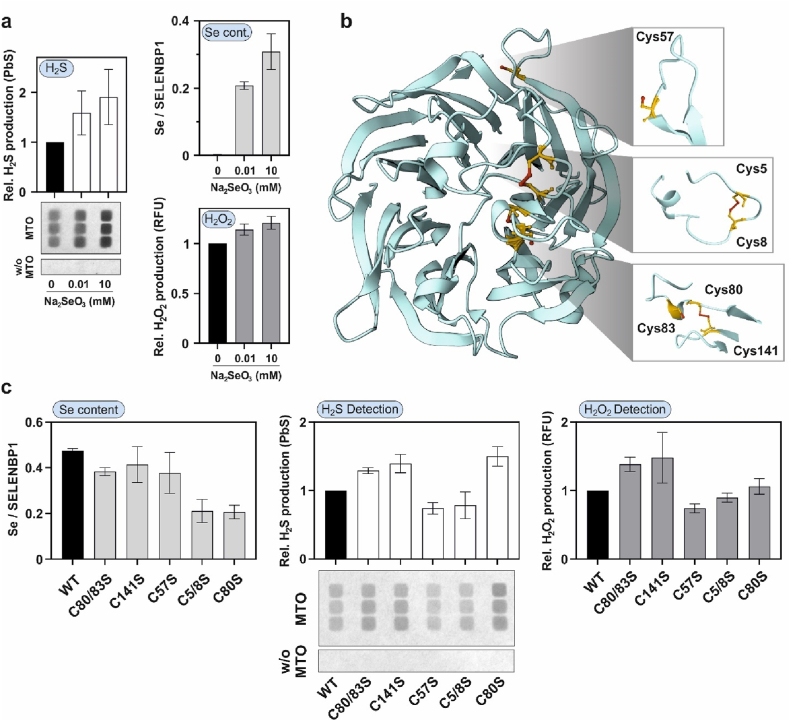
**Selenium binding by SELENBP1: effect on MTO activity and contribution of cysteine residues. a,** Recombinant Strep-tagged SELENBP1 was isolated from *E. coli* lysates and washed with sodium selenite during affinity chromatography at the indicated concentrations. The Se content of SELENBP1 was measured by total reflection X-ray fluorescence spectrometry (TXRF). MTO activity was determined by conversion of methanethiol, produced *in situ* through recombinant MGL, to H_2_S and H_2_O_2_. Data represent means ± SD from three independent experiments. **b,** Topology of candidate cysteine residues for Se binding in a 3D-model of SELENBP1 (UniProt-ID: Q13228) that is based on a prediction by the AlphaFold Protein Structure consortium. **c,** Wildtype or cysteine-deficient SELENBP1 (with the given cysteines replaced by serine) was washed with 10 mM selenite during affinity chromatography. Selenium content as well as methanethiol-derived H_2_S and H_2_O_2_ production were analyzed. Data represent means ± SD from three independent experiments, normalized to wildtype SELENBP1.

Here, we demonstrate that Cu is a required cofactor for the MTO activity of both SELENBP1 and SEMO-1, while Se is generally dispensable but may modulate their enzymatic function. Moreover, we show that the oxidoreductase activity of SELENBP1 is not restricted to its major substrate methanethiol but extends to structurally related alkyl thiols that may derive from secondary metabolites occurring in edible plants such as cabbage, garlic and onions.

## 2 Materials and methods

### 2.1 Reagents

Chemicals were purchased from Sigma-Aldrich (Munich, Germany) or Carl Roth (Karlsruhe, Germany), unless stated otherwise. Primers were obtained from Life Technologies (Darmstadt, Germany).

### 2.2 Cloning, *in-vitro* mutagenesis, bacterial overexpression and purification of recombinant proteins

The coding sequences of human SELENBP1 and *C. elegans* SEMO-1 were amplified by PCR, and plasmids for overexpression of the recombinant Strep-tagged proteins were generated as previously described [8,9]. An expression plasmid for recombinant Strep-tagged methionine gamma-lyase (MGL) from *Brevibacterium aurantiacum* was obtained as described [9]. Generation of single amino acid mutants of SELENBP1 and SEMO-1 occurred through site-directed *in-vitro* mutagenesis as described [8,9].

For bacterial overexpression of the recombinant wildtype and mutant proteins, *E. coli* strains BL21 (New England Biolabs, Ipswich, MA) and KRX (Promega, Madison, WI) were transformed with the respective plasmids. Bacteria were grown at 37 °C and 200 rpm in Luria/Miller LB medium (Carl Roth) supplemented with 50 μg/ml carbenicillin (Carl Roth) until they reached an OD (600 nm) of 0.6–0.8. Temperature was then lowered to 22 °C, and biosynthesis of recombinant proteins was induced in the BL21 strain by adding isopropyl-β-d-1-thiogalactopyranoside (IPTG; Serva Electrophoresis, Heidelberg, Germany) to a final concentration of 0.6 mM and in the KRX strain by adding rhamnose (Carbolution Chemicals, St. Ingbert, Germany) to a final concentration of 0.06% (w/v). Bacteria were incubated for 3 h in the presence of the inducers and then harvested by centrifugation (6000×*g*; 4 °C; 5 min); the obtained pellets were frozen at −80 °C. For isolation of the Strep-tagged recombinant proteins, bacteria were resuspended in HEPES-buffered saline (HBS; 50 mM HEPES, 150 mM NaCl, pH 7.4) and lysed by sonication (58% output; 10 s pulse; 10 times). The soluble fraction was separated by centrifugation (4 °C; 16000×*g*; 15 min) and transferred to a Strep-Tactin®XT 4Flow® column (IBA Lifesciences, Göttingen, Germany). Protein purification was accomplished according to the manufacturer's protocol. To remove contaminating proteins, three more washing steps with 1 ml HBS containing 20 mM MgCl_2_ and 5 mM ATP were added. If applicable, treatment of proteins with EDTA (10 mM), sodium selenite (10 μM, 10 mM) or copper (II)-chloride (10 μM) was then achieved by washing with 4 ml of the respective metal(loid)-containing solution (in HBS) and subsequent washing (10 times with 2 ml HBS). Recombinant proteins were eluted using HBS containing 50 mM biotin (IBA Lifesciences). Buffer change of the protein solution was accomplished through ultrafiltration using a Vivaspin ultrafiltration column with a cut-off of 10 kDa (Sartorius, Göttingen, Germany). Protein solutions were stored at −80 °C in aliquots to avoid multiple freeze/thaw cycles and enzyme activity loss.

### 2.3 Maintenance and treatment of *C. elegans*

The wildtype Bristol N2 strain of *C. elegans* and the *E. coli* OP50 strain were obtained from CGC (Caenorhabditis Genetics Center, 10.13039/100007249University of Minnesota), which is supported by the National Institutes of Health-Office of Research Infrastructure Programs. The SEMO-1-deficient strain PHX2078, *semo-1(syb2078)*, was generated by SunyBiotech (Fuzhou, China) and outcrossed twice into N2 wildtype worms in our laboratory before use [8]. *C. elegans* overexpressing pY37A1B.5::Y37A1B.5::gfp was previously generated as described [14]. Nematodes were maintained as described [8,14], at 20 °C on nematode growth medium (NGM) agar plates spotted with *E. coli* OP50. In brief, synchronized L1 larvae were transferred to NGM agar plates and grown for 72 h to adulthood. Thereafter, the nematodes were washed off the plates with S-basal buffer and transferred to NGM agar treatment plates spotted with heat-inactivated (45 min; 65 °C) bacteria and supplemented with Na_2_SeO_3_ or CuCl_2_ to a final concentration of 0.1 mM or the copper chelator bathocuproine disulfonate (BCS) to a final concentration of 1 mM. After 24 h, the worms were collected and frozen at −80 °C. Frozen worm pellets were weighed, transferred into screw tube vessels and diluted 1:1 (m/v) with lysis buffer [100 mM Tris, 150 mM NaCl, pH 9.9, containing 1% (v/v) EDTA-free protease inhibitor cocktail (Carl Roth 3751.1)]. Pellets were lysed in three cycles in a tissue lyser with 30 Hz and then sonicated in 3 cycles (20% output; 10 s pulse; 5 times) on ice. The protein fraction was separated by centrifugation (4 °C; 20 min; 12000×*g*), and protein concentrations were measured using a standard bicinchoninic acid (BCA) assay.

### 2.4 Determination of MTO activity

Methanethiol oxidase (MTO) activity in samples was measured using a coupled enzymatic assay, with recombinant methionine gamma-lyase (MGL) as source of methanethiol as previously described [8,9]. In brief, MTO reaction mixes were prepared in 384-well plates in a total volume of 40 μl/well, containing potentially MTO-active samples (isolated recombinant proteins and *C. elegans* lysates at final protein concentrations of 0.1625 mg/ml and 1 mg/ml, respectively). The MTO reaction mixes were set up as triplicates in the vicinity of wells containing MGL reaction mixes. The MGL reaction was started by adding substrate solution (l-methionine in HBS) to MGL solution (MGL in HBS), yielding final concentrations of 20 mM methionine and 0.225 mg/ml MGL. Any metal cations were added to MTO reaction mixes as chloride salts, dissolved in HBS. Negative controls, where the MGL reaction was initiated without methionine in the reaction mixes, were set up on a separate 384-well plate. After starting the MGL reaction, the plate was quickly covered by an indicator paper, previously soaked in 20 mM Pb(II)-acetate, for detection of H_2_S generation. The enzymatic reactions took place at 37 °C and under shaking at 150 rpm for 3 h. Thereafter, PbS spots on the indicator paper were detected with a ChemiDoc MP analyzer and Image Lab software (Bio-Rad Laboratories; Munich, Germany) to assess MTO-generated H_2_S. The H_2_O_2_ produced by the MTO reaction was measured using the Fluorometric Hydrogen Peroxide Assay Kit (Sigma Aldrich) at ʎ_ex_ = 540 nm and ʎ_em_ = 590 nm in a microplate reader (CLARIOstar; BMG Labtech; Ortenberg, Germany).

To test for additional substrates of SELENBP1 and SEMO-1, the volatile substances methanethiol, ethanethiol, H_2_S and methaneselenol (boiling points: 6 °C, 35 °C, −60 °C, 12 °C) were generated by MGL-catalyzed degradation of l-methionine, dl-ethionine, l-homocysteine or dl-selenomethionine, respectively. All reaction wells were covered with Pb(II)-acetate-soaked indicator paper except for the substrates H_2_S and methaneselenol, for which untreated filter paper was used due to direct reactions of these compounds with Pb(II)-acetate. In parallel to the MTO reaction, generation of volatile substrates was measured photometrically with Ellman's reagent, DTNB (5,5′-dithiobis-2-nitrobenzoic acid; Carl Roth). 40 μl/well of DTNB solution (50 mM in HBS; pH 7.4) was placed in close proximity to the MGL reaction vessel in triplicates (see Fig. 4a). After 3 h of incubation, DTNB solution was diluted 1:100 in 0.1 M potassium phosphate buffer containing 1 mM EDTA (pH 8.0), and absorbance was measured at 412 nm as described [9]. Non-volatile chemicals were dissolved or diluted in HBS and added to the MTO reaction well directly to yield a final concentration of 100 μM. Assessment of H_2_S and H_2_O_2_ were performed as described above.

### 2.5 Trace element analysis

Cu and Se content of recombinant wildtype and mutant SELENBP1 was analyzed by total reflection X-ray fluorescence (TXRF) spectrometry, using a bench-top TXRF spectrometer (S4 T-STAR, Bruker Nano, Berlin, Germany) as described [18]. Wildtype and mutant proteins were diluted with HBS to a concentration of 7.5 μM (420 mg/L) and combined with 1 mg/L Yttrium (Merck/Millipore, Darmstadt, Germany) as internal standard. 10 μl of each sample were placed on a siliconized sample carrier and dried at 40 °C. All samples were measured in duplicates for 1000 s each.

### 2.6 Prediction and modelling of SELENBP1 and SEMO-1 protein structures

A PDB file of human SELENBP1 (UniProt-ID: Q13228) was downloaded from AlphaFold Protein Structure Database (https://alphafold.ebi.ac.uk), a product of DeepMind and EMBL's European Bioinformatics Institute (EMBL-EBI), and verified by comparison to structure homology-modelling server SWISS-MODEL [[19], [20], [21]]. Modelling and visual adjustments, for demonstration purposes only, were performed using the standalone version of the Mol* 3D viewer of RCSB Protein Data Bank (PDB). Putative binding sites were identified by analysis of predicted structures and utilizing the COFACTOR program based on functional and homologous binding sites from the BioLiP database [22].

### 2.7 Statistical analysis

Data are expressed as means ± SD, unless stated otherwise. All calculations were performed using GraphPad Prism (GraphPad Software, San Diego, CA).

## 3 Results

### 3.1 Selenium binding only moderately supports, and is not required for, the MTO activity of SELENBP1

SELENBP1 was named based on its Se-binding capability, which required the presence of high doses of selenite [2]. On the other hand, SELENBP1 was devoid of Se in the liver of healthy rats, but Se attached to SELENBP1 after streptozotocin treatment that resulted in development of diabetes [23]. This led to the assumption that binding of Se to SELENBP1 may occur under non-physiological and/or stressed conditions, such as in Se toxicity [24]. Consistently, recombinant human SELENBP1 produced in *E. coli* did not contain detectable Se when no selenite was added during purification (Fig. 1a). Despite being devoid of Se, recombinant SELENBP1 was enzymatically active: MTO activity was assessed using an *in vitro*-assay [9] through both H_2_S and H_2_O_2_ release (Fig. 1a). Exposure of recombinant SELENBP1 to selenite resulted in dose-dependent association of Se to the recombinant protein. However, even washing freshly isolated SELENBP1 with buffer containing very high doses of selenite (up to 10 mM) did not result in a 1:1 stoichiometric ratio of Se and SELENBP1. Moreover, MTO activity of SELENBP1 only moderately increased following exposure to selenite, implying that Se was dispensable for MTO activity of SELENBP1 (Fig. 1a).

It was hypothesized earlier that Se binds covalently to SELENBP1 through a seleno-sulfide bond [3]. Among the ten cysteine residues in human SELENBP1, Cys^57^ was postulated as the putative Se-binding site, based on considerations of structural requirements and on molecular dynamics studies [17]. However, Cys^57^ is missing in many SELENBP1 orthologs, disqualifying this residue as the exclusive site for Se-binding. In the *Arabidopsis thaliana* ortholog of SELENBP1, Cys^21^ and Cys^22^ were identified to bind Se within a R-S-Se-S-R structure [15]. Human SELENBP1 has an N-terminal CXXC (Cys^5^-X-X-Cys^8^) motif that we hypothesized to contribute to Se binding as well: based on structural considerations, Cys^5^/Cys^8^ as well as Cys^80^/Cys^141^ represent two pairs of close cysteine residues potentially capable of interacting with selenite to form a R-S-Se-S-R bond; these cysteine pairs may also form intramolecular disulfide bonds (Fig. 1b). In order to explore the relevance of the candidate cysteine residues for Se-binding and MTO activity, we generated a series of SELENBP1 mutants with cysteines replaced by serines.

Se binding was decreased for the Cys^5,8^Ser and the Cys^80^Ser mutants compared to wildtype SELENBP1, suggesting involvement of the Cys^5^/Cys^8^ as well as Cys^80^/Cys^141^ pairs in Se binding. However, the latter is rendered an unlikely contributor as the Cys^80,83^Ser and Cys^141^Ser mutants showed no major alterations in Se binding (Fig. 1c). Regarding MTO activity, no link to changes in Se binding was observed: the Cys^80,83^Ser, Cys^80^Ser and Cys^141^Ser mutants showed a slightly higher activity than wildtype SELENBP1. This might point to a contribution of a Cys^80^/Cys^141^ disulfide bond to the regulation of accessibility of the SELENBP1 catalytic site by stabilizing the tertiary structure. In contrast, the MTO activity of the Cys^5,8^Ser mutant was not different from wildtype SELENBP1 (Fig. 1c).

As mentioned above, Cys^57^ was postulated to play a major role in SELENBP1 biological activity and selenium binding [17]. Here, however, no difference in Se binding or MTO activity from wildtype SELENBP1 was found for the Cys^57^Ser mutant (Fig. 1c), supporting the notion that this cysteine residue is not a major determinant of either Se binding or MTO activity of SELENBP1.

In summary, MTO activity of SELENBP1 is largely independent of Se binding. Moreover, Se binding of SELENBP1 is not mediated through Cys^57^, but primarily through Cys^5,8^.

### 3.2 SELENBP1 and its *C. elegans* ortholog SEMO-1 are copper-dependent MTOs

When establishing the isolation of recombinant SELENBP1 in order to test for its MTO activity, we noted that differences in EDTA content of the buffers applied for isolation resulted in different MTO activity outcomes. Therefore, we asked whether metal ions might be required for the MTO activity of SELENBP1 and also its *C. elegans* ortholog SEMO-1 that we had previously identified [8,14]. Literature reports supported the hypothesis that these proteins may be metal-dependent enzymes: bacterial [13] and plant [15] orthologs of SELENBP1 were previously demonstrated to be capable of binding several divalent metal ions, and the rat ortholog, Selenbp1, showed up in a metalloproteome analysis of metal binding proteins in diabetic rats [23].

**Fig. 2:**
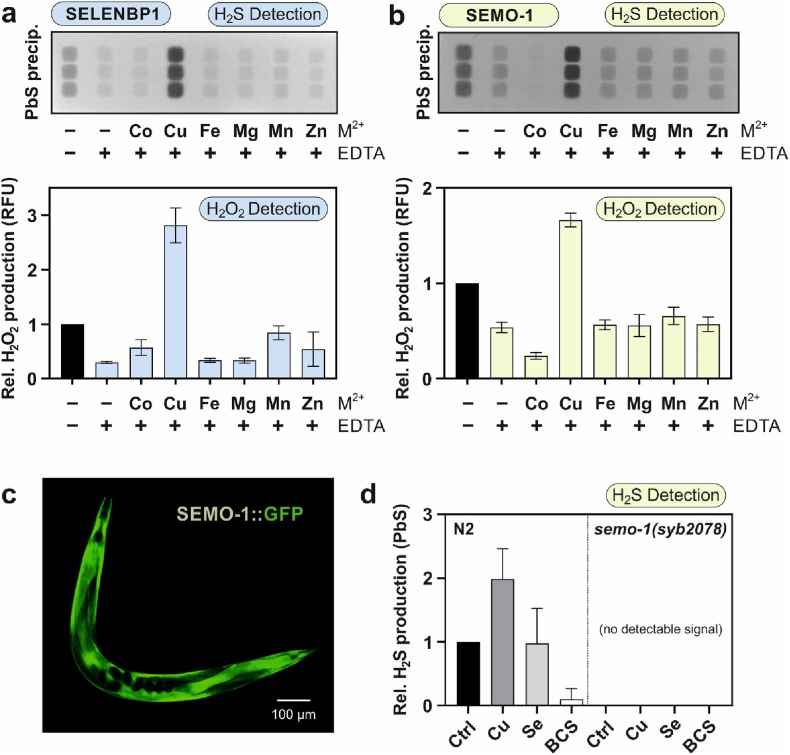
**MTO activity of SELENBP1 and SEMO-1 is copper-dependent. a, b,** Recombinant Strep-tagged SELENBP1 and SEMO-1 were produced in *E. coli* and washed (pre-treated) with 10 mM EDTA during affinity chromatography where indicated. Thereafter, the given divalent cations were supplemented as chloride salts dissolved in HBS to a final concentration of 10 μM (1:2 protein/cation). MTO activity was determined with MGL-produced methanethiol as substrate. **a,** MTO activity of SELENBP1, as assessed using H_2_S release (upper panel) and H_2_O_2_ production (lower panel) as readouts. **b,** MTO activity of SEMO-1. **c,** SEMO-1 localization in *C. elegans*, as visualized by fluorescence microscopy of a worm carrying a SEMO-1::GFP translational reporter. **d,** Relative methanethiol-derived H_2_S production (as detected by lead acetate indicator paper) in lysates from wildtype (N2) and SEMO-1-knockout worms (right panel). Synchronized worms were allowed to grow for 72 h to adulthood, and thereafter, they were treated for 24 h with 0.1 mM CuCl_2_, 0.1 mM sodium selenite or 1 mM of the copper chelator BCS. Data are normalized against the H_2_S production of non-treated wildtype worms and depicted as means +SD of three independent experiments.

Washing recombinant SELENBP1 (Fig. 2a) or SEMO-1 (Fig. 2b) with EDTA during purification decreased their MTO activity (detected through H_2_S and H_2_O_2_ generation; column/bar 2 *vs*. column/bar 1). In order to regenerate MTO activity, various divalent metal ions (Co^2+^, Cu^2+^, Fe^2+^, Mg^2+^, Mn^2+^, or Zn^2+^) were then added to the proteins to an approximately two-fold molar excess, followed by MTO activity analysis. As demonstrated in Fig. 2a/b, only the addition of cupric ions was capable of regenerating and even enhancing MTO activity of SELENBP1 and SEMO-1, whereas none of the other tested metal ions were able to reconstitute MTO activity. Thus, SELENBP1 and SEMO-1 require copper as a cofactor for their enzymatic activity, similar to a bacterial SELENBP1 ortholog from *Hyphomicrobium* sp., in which two binuclear copper centers were hypothesized to be present [13].

Having established the equivalence of SELENBP1 and SEMO-1 with respect to MTO activity being copper-dependent, we used *C. elegans* to assess the significance of copper availability for MTO activity in an *in vivo* model. In adult *C. elegans*, SEMO-1 is localized predominantly to the hypodermis, as seen in worms expressing GFP-labeled SEMO-1 [8,14](Fig. 2c). To assess whether copper bioavailability may affect MTO activity *in vivo*, worms were grown on agar supplemented with CuCl_2_ or with the Cu chelator bathocuproine disulfonate (BCS) for 24 h, followed by lysis and analysis of MTO activity.

MTO activity in lysates of wildtype worms grown on Cu-supplemented agar was increased by 2-fold over control conditions, as assessed by methanethiol-dependent H_2_S generation (Fig. 2d). This suggests that SEMO-1 is not Cu-saturated in nematodes kept under laboratory conditions. However, this might differ when the worms live in their natural habitat, soil, which is subject to large geographical variation in copper content [25]. Growth in the presence of BCS, on the other hand, resulted in a downregulation of MTO activity by approximately 90% (Fig. 2d). As in humans, copper is taken up by cells in *C. elegans* in the cuprous form, most likely through CHCA-1, an ortholog of the human copper transporter CTR1 [26]. Exposure to selenite did not affect methanethiol-derived H_2_S production in lysates of wildtype worms, in line with Se not being a prerequisite for MTO activity of SELENBP1 and its orthologs. No MTO activity was detected in lysates of SEMO-1 knockout worms, independent of Cu or Se supply (Fig. 2d).

Taken together, these data show that Cu is an indispensable cofactor for the MTO activity of SELENBP1 and SEMO-1.

### 3.3 Identification of SELENBP1 sites involved in Cu binding

**Fig. 3:**
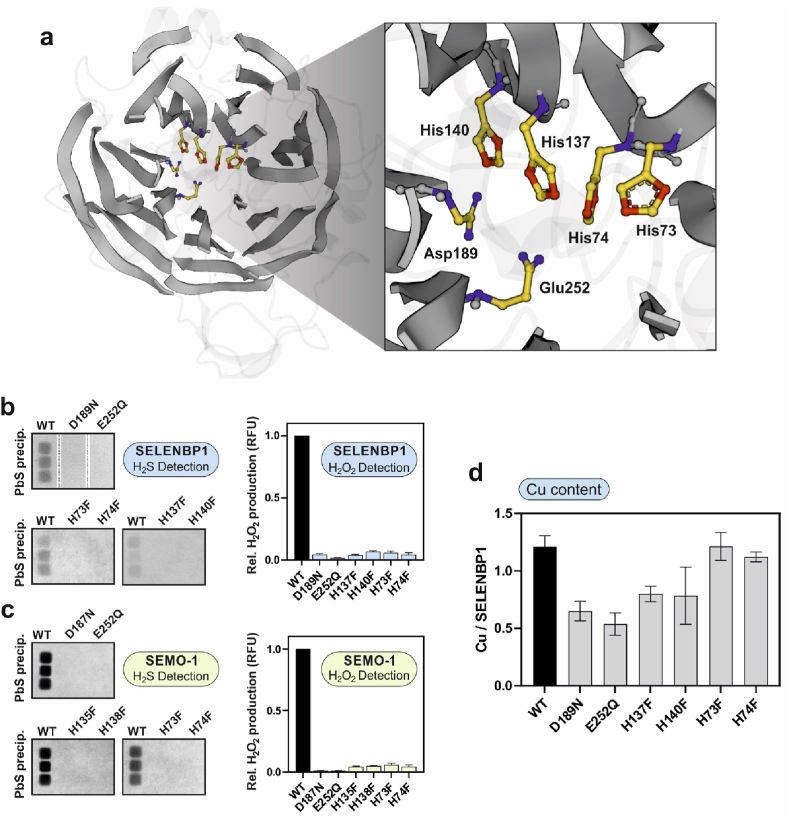
**Identification of Cu-binding amino acids in human SELENBP1, and evaluation of their relevance for its MTO activity. a,** 3D-model of SELENBP1 (AlphaFold), highlighting the β-propeller structure of the protein. Right panel: magnified cavity showing the modelled topological location of the amino acids proposed to mediate copper coordination (oxygen: blue; nitrogen: red; carbon: yellow). Putative Cu-binding amino acids and motifs were identified using the COFACTOR program. **b, c,** SELENBP1 and SEMO-1 mutants were generated through site-directed *in vitro*-mutagenesis. Recombinant wildtype and mutant proteins were produced in *E. coli* and treated with 10 μM CuCl_2_ during affinity purification; excess Cu^2+^ was removed through subsequent washing with HBS. MTO activity was tested assessing the release of H_2_S and H_2_O_2_ from enzymatically generated methanethiol and copper content of the proteins was determined using TXRF; three independent experiments were performed. **d,** Copper content of the wildtype and mutant recombinant SELENBP1 proteins. (For interpretation of the references to colour in this figure legend, the reader is referred to the Web version of this article.)

No SELENBP1 crystal or solution structure is available to date. However, modelling based on the X-ray template structure of the SELENBP1 ortholog from *Sulfolobus tokodaii* (PDB ID: 2ECE) and an artificial intelligence-supported approach based on amino acid sequences [19] revealed a predicted globular tertiary structure of the protein, consisting mainly of β-sheets with a few α-helices and disordered regions (Fig. 3a). In this predicted model, SELENBP1 contains seven highly symmetrical, toroidally arranged β-sheets that establish a cavity along the central axis. Each β-sheet is formed by four anti-parallel β-strands. The β-sheets are connected by loops located at bottom and top of the β-propeller structure (Fig. 3a). The cavity of SELENBP1 in this model contains histidine residues that are part of a HXXH (His^137^-X-X-His^140^) and an XHHX motif (X-His^73^-His^74^-X), respectively, both discussed as putative metal-binding sites in SELENBP1 orthologues [15,27]. An aspartyl (D^189^) and a glutamyl (E^252^) residue are located in close topological proximity (Fig. 3a). Both Asp and Glu are often involved in binding divalent cations in combination with His residues; like the HXXH and XHHX motifs, they are conserved among multiple SELENBP1 orthologs. Based on proximity, accessibility and data from other metal-binding β-propeller proteins such as diisopropyl-fluorophosphatase from squid (UniProt ID: Q7SIG4), a bacterial nitrous-oxide reductase (UniProt ID: Q51705) and a fungal galactose oxidase (PDB: 1GOF), we hypothesized H^73^, H^74^, H^137^, H^140^, D^189^ and E^252^ to contribute to binding of copper ions in SELENBP1.

**Fig. 4:**
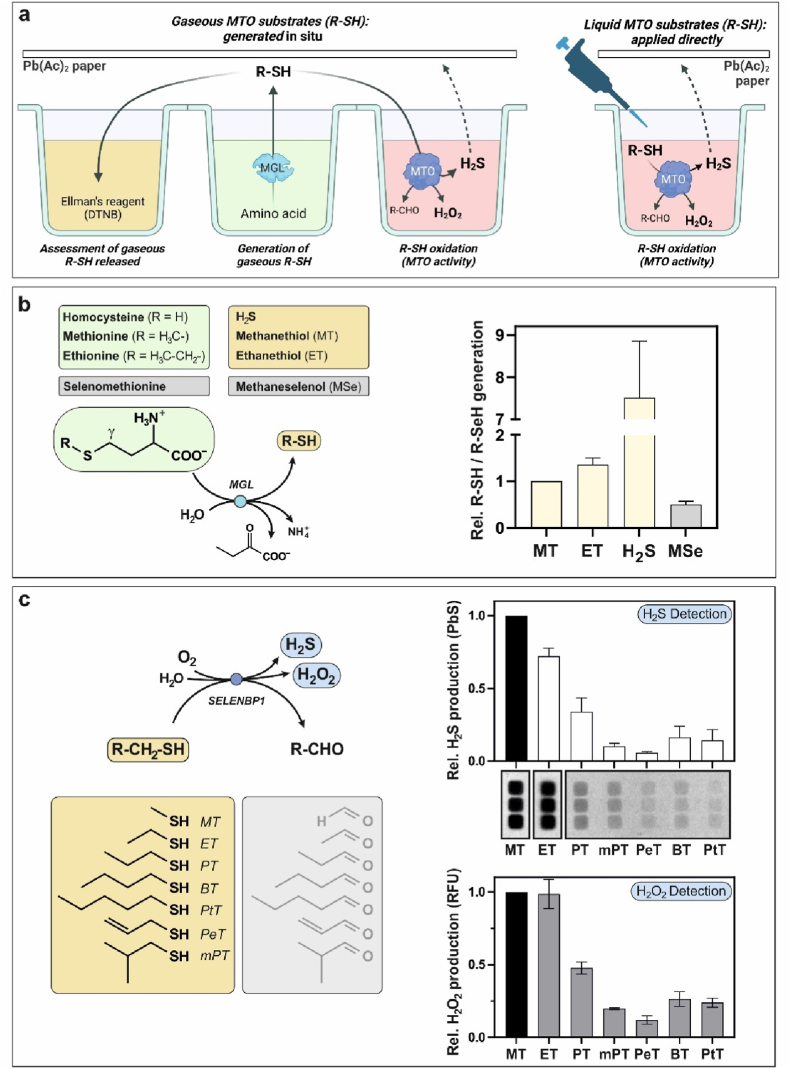
**SELENBP1 substrates other than methanethiol: SELENBP1 is a thiol oxidase. a,** Schematic drawing of the assay developed to test for the capability of SELENBP1 to convert different volatile and non-volatile substrates. The assay was set up in reaction wells in a 384 well-plate. Generation of potential gaseous SELENBP1 substrates through MGL-catalyzed elimination reactions was followed (in adjacent reaction wells) by assessment of the volatile thiols/selenols using Ellman's reagent with spectrophotometric measurement of TNB^2−^ at 412 nm, and by testing for thiol oxidase activity of recombinant SELENBP1 using the volatile substrates (left panel). Liquid substrates were added directly to the SELENBP1 reaction wells (right panel). This scheme was created with BioRender.com. **b,** MGL-catalyzed generation of volatile thiols/selenols, as detected using Ellman's reagent. **c,** Detection of SELENBP1-catalyzed H_2_S and H_2_O_2_ production from different substrates. Substrates tested and reaction scheme are given on the left. H_2_S release (upper right panel) was assessed through lead (II)-acetate indicator paper; image of the lead sulfide precipitates by three independent protein isolates and densitometric quantitation of pixel density are shown. H_2_O_2_ production (lower right) was quantitated through a fluorometric HRP-coupled assay. All data are given as relative means ± SD from three independent experiments; the standard SELENBP1 substrate methanethiol was set to 1. (MT: methanethiol, ET: ethanethiol, PT: propanethiol, mPT: 2-methyl-1-propanethiol, PeT: 2-propene-1-thiol, BT: butanethiol, PtT: pentanethiol).

In order to explore whether the above histidyl, aspartyl and glutamyl residues are required for copper binding and MTO activity of SELENBP1, mutants were generated by site-directed *in vitro*-mutagenesis: histidines were replaced by phenylalanine, while Asp and Glu were replaced by Asn and Gln, respectively. All mutants showed a strongly suppressed MTO activity relative to wildtype SELENBP1, with very little methanethiol-derived H_2_S and H_2_O_2_ generation (Fig. 3b), demonstrating the relevance of these six amino acids for the enzymatic function of the protein.

The same outcome was observed for SEMO-1: mutations of either of the analogous six amino acids (H^73^, H^74^, H^135^, H^138^, D^187^ and E^252^) caused an almost complete loss of MTO activity (Fig. 3c).

Analysis of copper content of wildtype SELENBP1 using total reflection X-ray fluorescence (TXRF) spectrometry indicated 1.2 Cu per 56 kDa-SELENBP1 molecule (Fig. 3d), suggesting more than one copper-binding site in SELENBP1. This is consistent with data previously published for the SELENBP1 ortholog from *Hyphomicrobium* sp., where a molar ratio of 1.4:1 (copper/protein) was calculated [13]. Interestingly, none of the mutants had completely lost the ability to bind Cu, despite an almost complete loss of MTO activity in each case; moreover, a decrease in copper-binding was observed only for the H^137^F, H^140^F, D^189^N and E^252^Q mutants, whereas the H^73^F and H^74^F mutants did not exhibit a copper content different from wildtype SELENBP1 (Fig. 3d). These data support the idea that, as multiple amino acid residues are involved in Cu binding, mutagenesis of one contributing amino acid may cause a decrease in Cu affinity but is unlikely to completely abolish Cu binding. The stability of isolated SELENBP1 or SEMO-1 was not affected by decreased copper-binding capability, as the mutant recombinant proteins showed no degradation or differences in SDS-PAGE gels and immunoblots, as compared to the wildtype protein (data not shown).

In summary, based on structure prediction and data on MTO activity and Cu content of SELENBP1, we suggest an important role of the proposed copper-binding pocket for MTO activity, with copper binding likely mediated through H^137^, H^140^, D^189^ and E^252^.

### 3.4 SELENBP1 is a thiol oxidase that accepts several thiols as substrates besides methanethiol

The enzymatic function of SELENBP1 has been attributed to oxidation of methanethiol derived from the metabolism of gut microbiota [7]. To assess whether SELENBP1 may catalyze the oxidation of structurally related sulfur and selenium compounds as well, we used other thiols (both volatile, such as ethanethiol, and non-volatile, i.e. liquid) as substrates. First, we tested whether MGL accepts substrates other than methionine, in order to generate potential gaseous substrates. In fact, ethanethiol, methaneselenol and H_2_S were generated by MGL-mediated elimination from their precursor amino acids, ethionine, selenomethionine and homocysteine, respectively (Fig. 4a and b). MGL-mediated generation of methanethiol from methionine served as positive control. Concentrations of the produced volatile substances were assessed by Ellman's reagent, DTNB, and calculated based on the extinction coefficient of thionitrobenzoate (Fig. 4b); they were then used as reference values for the non-volatile analytes that were added directly to SELENBP1 (Fig. 4a, right). SELENBP1-mediated H_2_S and H_2_O_2_ production was assessed, with heat-denatured protein used as negative control to normalize for auto-oxidation of thiols mediated by copper bound to SELENBP1.

Among the tested sulfur compounds, methanethiol (MT), ethanethiol (ET), 1-propanethiol (PT), 2-methyl-1-propanethiol (mPT), 2-propene-1-thiol (PeT), 1-butanethiol (BT) and 1-pentanethiol (PtT) produced sufficient amounts of H_2_S in the SELENBP1-catalyzed reaction for detection by Pb(II)-acetate indicator paper. On the other hand, H_2_O_2_ production, normalized for thiol autooxidation, was observed with all mentioned thiols (Fig. 4c). Fig. 4c lists the structures of the tested thiols. Structures of generated aldehydes are held in gray as they are hypothetical in that the only products we demonstrated were H_2_S and H_2_O_2_.

The chalcogens sulfur and selenium have similar physicochemical properties. Some enzymes such as MGL accept both sulfur- and the corresponding selenium-compounds as substrates [28]. Consistently, the recombinant bacterial MGL that we used in our assay was capable of producing methaneselenol from selenomethionine, although less efficiently than the sulfur-analogs (Fig. 4b). Interestingly, SELENBP1 was able to discriminate between methanethiol and methaneselenol, as indicated by the lack of H_2_O_2_ production when methaneselenol was tested as substrate (data not shown). This might be due to the lower pK_a_ of methaneselenol (pK_a_ ≈ 6, *vs.* methanethiol: pK_a_ ≈ 10.4) and its deprotonation at physiological pH.

In summary, SELENBP1 metabolizes several thiols, but methanethiol and ethanethiol are by far the best SELENBP1 substrates, as demonstrated by H_2_S and H_2_O_2_ production (Fig. 4c).

## 4 Discussion

Human SELENBP1 has been proposed to bind Se through its Cys^57^ residue, as this was thought to be the only freely accessible cysteine in the hydrophilic environment of the cytosol [17], where SELENBP1 is predominantly located [5]; however, this prediction was never proven experimentally. Nevertheless, HCT116 colon carcinoma cells overexpressing a Cys^57^Gly mutant of SELENBP1 were reported to be less protected against selenite toxicity than cells overexpressing the wildtype protein [29]. Here, we measured the Se content of selenite-treated wildtype and mutated recombinant SELENBP1 by TXRF, and while Se-binding was not suppressed in a Cys^57^Ser mutant, considerably less Se was associated to a Cys^5,8^Ser mutant (Fig. 1). This suggests that Se-binding of human SELENBP1 is predominantly dependent on its N-terminal Cys^5^/Cys^8^ motif; similarly, an N-terminal Cys^21^/Cys^22^ motif has been shown to mediate Se-binding in the SELENBP1 ortholog of *A. thaliana* [15]. Another insufficiently answered question relates to the physiological role of Se bound to SELENBP1 and its influence on the function of the protein. Human SELENBP1 as well as its orthologs in *A. thaliana* and *C. elegans* may provide a buffer against Se toxicity by binding excess selenite [15,29,30]. Moreover, physical interaction of SELENBP1 with the von Hippel-Lindau protein-interacting deubiquitinating enzyme 1 (VDU1) has been reported to be dependent on Se-binding of SELENBP1 [31]. Regarding the oxidoreductase activity of SELENBP1, we here found that Se-binding was not a prerequisite but exposure of the recombinant protein to high selenite concentrations resulted in a very moderate increase in methanethiol oxidation (Fig. 1). Interestingly, SEMO-1::GFP expression was upregulated in *C. elegans* exposed to selenite [30]. The fact that no increase in MTO activity following selenite exposure was seen here (Fig. 2d) suggests that Se is neither required for, nor a major stimulator of, MTO activity in *C. elegans*.

**Fig. 5:**
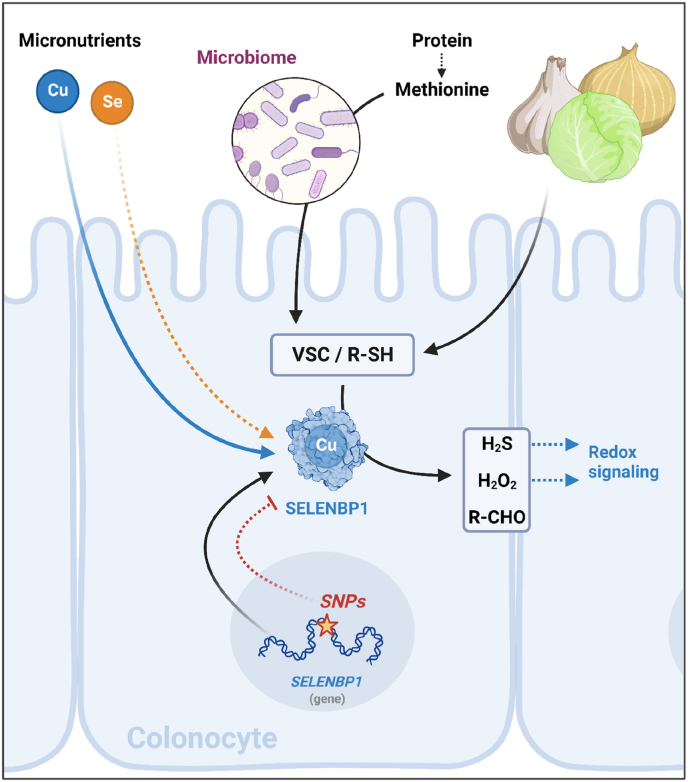
**Availability of dietary micronutrients (Cu and Se) is linked to oxidation of food-derived VSCs through the thiol oxidase activity of SELENBP1, resulting in the generation of redox signaling mediators.** See text for details. Created with BioRender.com.

Instead of Se, we identified copper as the cofactor required for the oxidoreductase activity of SELENBP1, and we mapped the involved amino acid residues in its postulated copper-binding pocket (Fig. 2, Fig. 3). Similarly, copper was required for SEMO-1, the SELENBP1 ortholog in *C. elegans*. The same was reported for the ortholog in *Hyphomicrobium* sp.; extended X-ray absorption fine structure (EXAFS) analysis of that protein indicated that copper in the resting enzyme was coordinated by four nitrogens (Cu–N), whereas fewer Cu–N bonds were observed upon exposure to its substrate methanethiol. In addition, alterations in oxidation state and coordination of copper were observed upon enzyme-substrate interaction [13]. Copper is known to interact with volatile sulfur compounds (VCSs), and it has higher affinity towards methanethiol than other divalent cations [32]. SELENBP1 is highly expressed in the colon, where it is localized predominantly in the epithelial cells at the top of the crypts that shape the interface to the colonic lumen [4,9]. The MTO activity of SELENBP1 was discovered when persons with extraoral halitosis were shown to carry bi-allelic mutations in the *SELENBP1* gene that resulted in a non-functional enzyme. As a result, they were not capable of metabolizing the methanethiol derived from colonic microbiota [7]. Interestingly, rinsing the mouth with CuCl_2_ has been shown to counteract halitosis by neutralizing VSCs [32]. We hypothesize that dietary copper might also aid the suppression of extraoral halitosis by supporting the MTO activity of SELENBP1 in the colon (Fig. 5).

We suggest H^137^, H^140^, D^189^ and E^252^ to be involved in SELENBP1 copper binding and to be essential for its oxidoreductase activity. Interestingly, corresponding polymorphisms in the human *SELENBP1* gene that result in alteration of some of these amino acid residues have been mapped previously, as listed in the Genome Aggregation Database (GnomAD) (see also Table 1); however, their effect on the MTO activity of the afflicted persons *in vivo* has remained elusive. We report here that site-directed mutagenesis of the above-mentioned amino acid residues resulted in complete loss of the MTO activity of these SELENBP1 mutants and a decrease in their copper content (Fig. 3). Given the extent to which the oxidoreductase activity was suppressed in these mutants *in vitro*, the occurrence of a phenotype of extraoral halitosis in individuals with the respective bi-allelic mutations seems likely. To date, suppression of MTO activity causing halitosis has been known to result from only two polymorphisms of the *SELENBP1* gene, G225W and H329Y [7,9]. Overexpression of recombinant SELENBP1 mutant proteins and exploration of their enzymatic activity *in vitro*, as done in this study, may thus contribute to better understanding potential functional consequences of genomic variants.

Finally, we found that the oxidoreductase activity of SELENBP1 is not limited to oxidation of methanethiol but extends to multiple structurally related alkyl thiols from different origins, mainly deriving from the catabolism of amino acids by gut bacteria and from ingested phytochemicals (Fig. 4, Fig. 5). The low olfactory threshold and the repulsive odor of alkyl thiols suggest an evolutionarily conserved conditioning of humans to avoid such substances. Methanethiol and ethanethiol, and to a lesser extent also propanethiol and butanethiol, exert toxic effects by inhibiting cytochrome *c* oxidase and thus impairing the mitochondrial respiratory chain [33,34]. Toxic effects of acute and chronic exposure to VSCs were investigated in a study with rats: The 24h LD_50_ value for acute doses of methanethiol was determined to be 675 ppm, while chronic exposure to sub-lethal doses of methanethiol resulted in decreased body weight of the rats [35]. Here, the question arises why methanethiol is converted by SELENBP1 to H_2_S, which has the highest affinity among the VSCs for cytochrome *c* oxidase and thus arguably a higher toxicity than methanethiol. Presumably, the availability of alternative metabolic pathways for the two substances might be important: methanethiol can be metabolized by secondary methylation to dimethyl sulfide, as catalyzed by the alkyl thiol methyltransferase activity of METTL7B; however, due to the low reaction rate, this occurs at an inadequate rate [11,36]. As shown in samples prepared from rat caecal mucosa, degradation of methanethiol via formation of H_2_S occurs orders of magnitude faster than its methylation [37]. H_2_S, on the other hand, can be utilized in the mitochondrial sulfide oxidation pathway, where it serves as an electron donor for ATP production in the respiratory chain [38]. Sulfide:quinone oxidoreductase (SQOR) catalyzes the first and committed step in this pathway that prevents the accumulation of toxic concentrations of H_2_S [39]. SQOR accepts different substrates that include low-molecular-mass thiols. Methanethiol was hypothesized to impede SQOR activity, ultimately interfering with its ability to metabolize H_2_S, when methanethiol concentrations are high [39]. Given the inhibition of SQOR by methanethiol, its rapid and efficient elimination is required to maintain H_2_S homeostasis and cellular respiration.

In addition to H_2_S, hydrogen peroxide and formaldehyde are being generated from methanethiol as catalyzed by SELENBP1. Whereas formaldehyde may be funneled into cellular C1 metabolism, both H_2_O_2_ and H_2_S are major cellular redox regulators, modulating cellular signaling events at multiple levels [[40], [41], [42]]. The fact that both products may also act on target proteins sequentially (H_2_O_2_ oxidizing Cys residues to form sulfenic acids, followed by interaction of H_2_S with the sulfenic acid), resulting in protein persulfidation [41], adds another level of complexity to the biology of SELENBP1. The exact contributions of SELENBP1 to cellular redox signaling, and the biological significance thereof, remain to be elucidated (Fig. 5).

## 5 Conclusions

SELENBP1 has long been known for its anti-proliferative actions as tumor suppressor that is down-regulated in many types of cancer [3]. In contrast, its recently discovered enzymatic (MTO) activity [7] and the relevance of essential trace elements as cofactors for this oxidoreductase function are less characterized. It is even disputable, whether the current designation “selenium-binding protein” is appropriate, as Se binding appears to require the presence of high non-physiological Se concentrations [24]. Here we showed that Se binding is basically dispensable for the MTO activity of SELENBP1. We characterized SELENBP1 as a protein that is dependent on copper as cofactor to be capable of catalyzing the oxidation of multiple primary alkyl thiols. The occurrence of SELENBP1 orthologs in all domains of life, in organisms that populate diverse habitats, implies a very early phylogenetic implementation of and need for the oxidation of alkyl thiols. In humans, highest levels of such VSCs occur in the colonic lumen, derived from gut bacteria and from ingested phytochemicals. Thus, the high expression level of SELENBP1 in the colonic epithelial cells may allow for rapid and efficient degradation of those compounds. SNPs in the SELENBP1 gene as well as the dietary supply with the essential micronutrients Cu and Se may affect the thiol oxidase activity of SELENBP1 (Fig. 5).

## Author contributions

Study conception and design: TMP, HS, LOK; acquisition of data: TMP, VAO, LG, MS, AW; analysis and interpretation of data: TMP, APK, HS, LOK; manuscript preparation: TMP, HS, LOK.

## Declaration of competing interest

None.

## Data Availability

Data will be made available on request.
